# Optimal cut-off value of waist circumference-to-height ratio to predict central obesity in children and adolescents: A systematic review and meta-analysis of diagnostic studies

**DOI:** 10.3389/fnut.2022.985319

**Published:** 2023-01-04

**Authors:** Maysa Eslami, Farzad Pourghazi, Maryam Khazdouz, Jing Tian, Kumars Pourrostami, Zahra Esmaeili-Abdar, Hanieh-Sadat Ejtahed, Mostafa Qorbani

**Affiliations:** ^1^Endocrinology and Metabolism Research Center, Endocrinology and Metabolism Clinical Sciences Institute, Tehran University of Medical Sciences, Tehran, Iran; ^2^Growth and Development Research Center, Tehran University of Medical Sciences, Tehran, Iran; ^3^Menzies Institute for Medical Research, University of Tasmania, Hobart, TAS, Australia; ^4^Social Determinants of Health Research Center, Alborz University of Medical Sciences, Karaj, Iran; ^5^Dietary Supplements and Probiotic Research Center, Alborz University of Medical Sciences, Karaj, Iran; ^6^Obesity and Eating Habits Research Center, Endocrinology and Metabolism Clinical Sciences Institute, Tehran University of Medical Sciences, Tehran, Iran; ^7^Non-Communicable Diseases Research Center, Alborz University of Medical Sciences, Karaj, Iran; ^8^Chronic Diseases Research Center, Endocrinology and Metabolism Population Sciences Institute, Tehran University of Medical Sciences, Tehran, Iran

**Keywords:** waist to height ratio, central obesity, abdominal obesity, children, adolescents

## Abstract

**Introduction:**

Waist circumference-to-height ratio (WHtR) is a simple anthropometric index with good screening power and fast interpretation for early detection of childhood abdominal obesity. This systematic review and meta-analysis aims to determine the best cut-off value of WHtR to use in clinical setting.

**Methods:**

Comprehensive searches were conducted in PubMed, Scopus, and Web of Science by the end of March 2021. Observational studies investigated the best WHtR cut-off to detect abdominal obesity in children and adolescents were included. Thirteen articles (*n* = 180,119) were included in this systematic review and eight documents were included in the meta-analysis.

**Results:**

The overall optimal cut-off was 0.49 with pooled sensitivity, specificity and diagnostic odds ratio (DOR) of 0.93 (95% confidence interval (CI): 0.93–0.96), 0.88 (95% CI: 0.85–0.91) and 102.6 (95% CI: 50.7–207.5), respectively. The optimal WHtR cut-off to predict abdominal obesity in girls and boys were both 0.49.

**Discussion:**

The current study shows that we could use this cut-off as a simple index for predicting abdominal obesity in children and adolescents without the need for any charts in practice.

## 1. Introduction

Nowadays, the increasing prevalence of childhood obesity has become a worldwide issue (1). Obesity has increased five times in children and adolescents since 1975 in the world, especially in developed countries (2). According to the latest the United Nations International Children’s Emergency Fund (UNICEF)/World Health Organization (WHO)/World Bank Group Joint Child Malnutrition Estimates, there are 38.3 million overweight children globally, an 8 million increase from 2000 to 2020 (2). Obesity is a multifactorial disease involving biological and environmental interactions such as physical activity, food consumption, sleep duration, etc. (3). Potentially, childhood obesity can lead to many devastating complications in adulthood, and central fat distribution is related to metabolic and cardiovascular diseases, particularly (1, 4).

It is helpful to identify an index with good screening power, easy measurement, and fast interpretation for early detection and management of childhood obesity (5). Body mass index (BMI) and waist circumference (WC) are the most commonly used measures for defining general and central obesity in clinical practices, respectively (6, 7). However, both measures are age, sex, and ethnicity dependent (8). After measuring WC or calculating BMI, both indices need standard growth charts matched for gender, age, and ethnicity, to assess the child’s anthropometric condition. It is well accepted that central obesity increases the risk for cardiometabolic disease in adults and children independent of general obesity measured by BMI, which cannot distinguish fat distribution (7). Although WC is strongly correlated with abdominal fat, it may over or under-evaluate the risk for the tall or short individual with the same WC (7) besides relying on age and sex-specific cut-off values (4).

In recent years, waist circumference-to-height ratio (WHtR) has been suggested as an alternative anthropometric indicator for screening central obesity. WHtR is a simple index without the need for age and gender-specific charts for interpretation (3). The National Institute for Health and Care Excellence (NICE) recommended WHtR as a simple index which could be measured by people themselves easily, and they can interpret the result whether they are at high health risk or not (9).

Prior studies found that WHtR is more sensitive, cheaper, and easier to measure and calculate than BMI and WC and can be used for both genders (10). Waist circumference changes with puberty, so we cannot propose the same cut-off for central obesity, but WHtR changes slightly with age, and its variations between boys and girls are not significant (11). A considerable number of studies have recommended using the WHtR cut-off value of 0.5 as a marker for screening central obesity in children and adolescents (7) with a simple health message “keep your waist circumference to less than half of your height” (12). However, some studies showed different thresholds with more sensitivity and specificity in various ethnicities (11, 13–20). A recently published meta-analysis reported different WHtR cut-offs for different regions as a screening tool for cardiometabolic risks in children and adolescents (1, 21).

Although many studies have investigated the utility of the WHtR as an index to screen central obesity and cardiometabolic risk among children and adolescents, there has been no systematic approach to identify a pooled cut-off of WHtR. The NICE committee noted that WHtR is the best measure for central obesity, but the evidence identified on boundary values for children and adolescents is not as sufficient as the evidence for adults. Considering that it is necessary to invoke a cut-off or boundary value for an index to use in public health for screening (7), the purpose of this systematic review and meta-analysis is to sum up the evidence and to assist NICE by finding the best cut-off value with high sensitivity and specificity for WHtR, a simple and easy indicator to screen central obesity in children and adolescents.

## 2. Materials and methods

**FIGURE 1:**
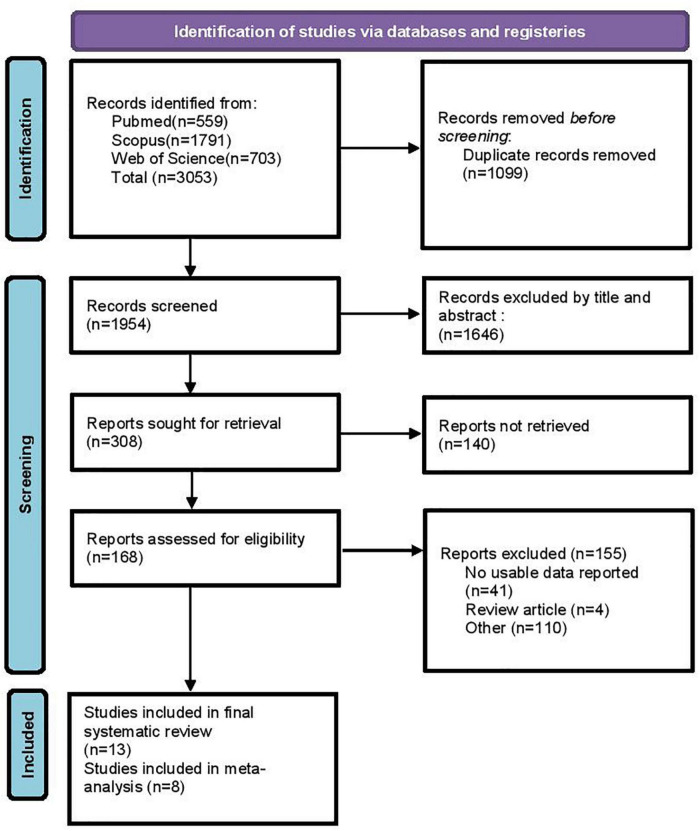
Flow chart for study identification and selection. Based on PRISMA 2020.

This systematic review and meta-analysis followed the established guidelines from the Preferred Reporting Items for Systematic Reviews and Meta Analyses (PRISMA statements) (Figure 1) (22).

### 2.1. Search strategy

Electronic searches were conducted in three major databases: PubMed, Scopus, and Web of Science. The search strategy included search terms for “pediatrics” OR “children” OR “adolescents” OR “students” AND “WHR” OR “waist to height ratio” OR “waist-height ratio” AND “abdominal obesity” OR “central obesity” OR “visceral obesity” OR “abdominal adiposity” OR “central adiposity” OR “abdominal fat” OR “Central fat.” Searches were limited to studies published in English by the end of September 2022. The reference list of included articles was also screened.

### 2.2. Eligibility criteria

We included all the cross-sectional original articles reporting a cut-off value for WHtR in children and adolescents to detect central obesity (with reporting sensitivity and specificity).

Articles were excluded if they did not evaluate central obesity or if their study population was adult. Clinical trials, review articles, conference proceedings, and book chapters also were excluded. Moreover, we excluded the studies that calculated WHtR diagnostic ability to predict central obesity in children and adolescents according to a predefined cut-off value.

### 2.3. Data collection

#### 2.3.1. Selection of studies

After the electronic search, all records were imported into Endnote software version X8, and duplicates were removed. Two researchers independently reviewed all articles based on titles and abstracts, then full-text of the included studies were judged and reviewed by inclusion criteria. Any disagreement between the two researchers was resolved by discussion until reaching consensus. A total of thirteen articles met the inclusion criteria for this systematic review (3, 11, 13, 20, 23–31). E-mails were sent to corresponding authors for any supplementary data. The studies selection process is summarized in the PRISMA flow diagram (Figure 1).

#### 2.3.2. Data extraction

Data were extracted independently from included articles by two authors according to predefined data extraction sheet. The extracted data included:

(1) General information (authors, publication year, country, study design).
(2) Participants’ characteristics (sample size, target population, age range).
(3) Diagnostic test for abdominal obesity.
(4) Cut-off values, sensitivity, specificity, the area under the curve (AUC), positive predictive value (PPV), negative predictive value (NPV).

### 2.4. Study quality assessment

The adapted Newcastle-Ottawa Quality Assessment Scale (NOQAS) for cross-sectional studies (32) was used to appraise the methodological quality of included papers. This scale consists of seven items within three categories including selection of participants (maximum 5 score), comparability of outcomes (maximum 2 score), and assessment of outcomes (maximum 2 score). The total score which ranges from 0 to 10 is the sum of all the scores. A higher score indicates lower risk of bias. We categorized the quality assessments as follows: 0 to 4 as “unsatisfactory,” 5 and 6 as “satisfactory,” 7 and 8 as “good,” and 9 and 10 points as “very good.” Two independent investigators conducted the quality assessment and a third investigator resolved any probable discrepancies.

### 2.5. Statistical analysis

We carried out a diagnostic test accuracy meta-analysis using a bivariate random-effects model. In the meta-analysis, we calculated the combined sensitivity, specificity, positive likelihood ratio (PLR), negative likelihood ratio (NLR), diagnostic odds ratio (DOR) and their 95% confidence intervals (CI), as summary estimates of cut-off scores accuracy based on the 2 × 2 tables (values of true positive, true negative, false positive, and false negative). Additionally, summary receiver operator characteristic (SROC) curves were created to assay the association between sensitivity and specificity. The heterogeneity was evaluated according to the *I*^2^-statistic of the pooled DOR. To find optimal cut-off score of WHtR, we performed meta-regression analysis and summarize operating sensitivity and specificity based on SROC curve. Since included studies have provided raw data of cut-off scores in the overall population and by sex, we decided to compute the optimal cut-offs into three categories: the overall optimal cut-off score, cut-off score in girls, and cut-off score in boys. We carried out a sensitivity analyses by excluding study that solely conducted in 3–5 years children or children under 10 years. Publication bias was evaluated based on Deek’s funnel plot analysis. When the *P*-value < 0.05, significant publication bias was considered. STATA 16.0 was used for statistical analysis.

## 3. Results

### 3.1. Literature research

Electronic searches in three databases retrieved 3,053 papers, of which 1,099 were duplicates. The remaining 1,954 papers were screened on titles and abstracts. After excluding 1,786 irrelevant papers, 168 full texts were reviewed, and 155 studies were further identified as ineligible. Finally, 13 articles were included in this systematic review (Figure 1). We could not pool data from 5 articles in the meta-analyses because of a lack of data.

### 3.2. Study characteristics

The general characteristics of the included studies are shown in Table 1. All the papers were cross-sectional in study design and published between 2008 and 2019. Studies originated from nine countries consisting of Brazil (three studies), Korea and China (two studies from each country), Pakistan, Turkey, Iran, Japan, Argentina, New Zealand (one study). Most of the studies were performed in Asia (*n* = 8). The sample size ranged from 108 to 121,025, yielding a total sample of 180,119 in our systematic review. The minimum and maximum age of participants was two and nineteen years old.

**TABLE 1:** General characteristics of included studies.

Number	Study	Country	Study design	Population	Sample size	Diagnostic test of abdominal obesity
1	Asif et al. (23)	Pakistan	Cross-sectional	5–12 years old public places and from public and private schools (primary, secondary and higher secondary)	T:5964 B:2865 G:3099	WC ≥ 90 Percentile
2	Carvalho et al. (24)	Brazil	Cross-sectional	10–18 years old adolescents of public school	T:731 B:252 G:479	Body fat with DXA
3	Choi et al. (11)	Korea	Cross-sectional	10–19 years old adolescents 15th Korean national survey	T:3057 B:1625 G:1432	WC ≥ 90 Percentile
4	Filgueiras et al. (25)	Brazil	Cross-sectional	4–9 years old children Born in Maternity hospital	T:788 B:407 G:388	Android Fat ≥ 90 percentile by DXA
5	Dong et al. (26)	China	Cross-sectional	7–18 years old Chinese National Survey	T:121025 B:60435 G:60590	WC ≥ 90 Percentile
6	Ejtahed et al. (27)	Iran	Cross-sectional	7–18 years old National school-based surveillance study	T:14274 B:7223 G:7051	WC ≥ 90 Percentile
7	Fujita et al. (28)	Japan	Cross-sectional	10 years old (Fifth grade) school children	T:466 B:226 G:196	Body fat with DXA
8	Guntsche et al. (29)	Argentina	Cross-sectional	6–16 years obese children and their siblings	T:108 B:NR G:NR	Trunk fat mass with DXA
9	Kilinc et al. (3)	Turkey	Cross-sectional	6–17 years old primary school/high school students	T: 2718 B:1467 G:1251	WC ≥ 90 Percentile
10	Kim et al. (30)	Korea	Cross-sectional	6–18 years old Korea National health and nutrition examination survey	T:13257 B:6987 G:6270	WC ≥ 90 Percentile
11	Sousa et al. (20)	Brazil	Cross-sectional	10–19 years old public School Children	T:516 B:152 G:364	Body fat with DXA
12	Taylor et al. (31)	New Zealand	Cross-sectional	3–5 years old predominantly white children	T: 301 B:151 G:150	Body fat with DXA
13	Zhou et al. (13)	China	Cross-sectional	7–17 years old children and adolescents from 6 regions of China	T:16914 B: 8843 G:8071	Chinese National Reference

T, total; B, boys; G, girls.

### 3.3. Quality assessments

The overall quality assessment of included studies ranged from 5 to 10. Most of the studies had 6 to 7 points thus falling within the “satisfactory” to “good” subgroups. The quality assessment results are summarized in Table 1.

### 3.4. General findings of the included studies

The reported cut-off values, sensitivity, specificity, AUC, and diagnostic test of abdominal obesity are summarized in Table 2. The Diagnostic test for evaluating central obesity was WC percentile or dual-energy X-ray absorptiometry (DXA). Among thirteen studies, seven have reported overall optimum cut-off, and nine articles have reported cut-off values for boys and girls, separately. The maximum cut-off point was 0.54 (29), and the minimum was 0.45 for both boys and girls (20). Almost all AUCs values in studies were close to 1. The highest and the lowest reported AUC (0.990 and 0.79) was for 0.50 Cut-off value (11, 31).

**TABLE 2:** The list of included studies with reported cut-offs and ROC curve analysis data for predicting abdominal obesity.

Number	References	AUC	Cut off points	Sensitivity (%) overall	Specificity (%) overall	PPV (%)	NPV (%)
1	Asif et al. (23)	B:0.969 (0.959–0.979)	B:0.47	B:97%	B:84%	NR	NR
		G:0.948 (0.936–0.961)	G:0.48	G:88%	G:87%		
2	Carvalho et al. (24)	B:0.98 (0.96–1.00)	B:0.46	B:93.6%	B:94.1%	NR	NR
		G:0.98 (0.96–0.98)	G: 0.48	G:90.2%	G:92.7%		
3	Choi et al. (11)	B:0.990	B:0.50	B:97.5%	B:94.4%	NR	NR
		G:0.985	G:0.48	G:94.6%	G:94.6%		
		T:0.978	T:0.48	T:96.4%	T:90.6%		
4	Filgueiras et al. (25)	B (4–5 y):0.904 (0.830–0.954) B(6–7 y):0.980(0.937–0.997)	B(4–5 y):0.51 B(6–7 y):0.51	B(4–5 y):90% (55.5–98.3)	B(4–5 y):89.1% (80.9–94.7)	B: (4–5 y):47.4	B: (4–5 y):98.8
		B(8–9 y):0.963(0.924–0.985) G(4–5 y):0.902(0.815–0.957)	B(8–9 y):0.49 G(4–5 y):0.50	B(6–7 y):100% (73.4–100)	B(6–7 y):91.8% (85–96.2)	(6–7 y):57.1 (8–9 y):53.1	(6–7 y):100 (8–9 y):99.3
		G(6–7 y):0.835(0.749–0.900) G(8–9 y):0.937(0.893–0.966)	G(6–7 y):0.50 G(8–9 y):0.47	B(8–9 y):94% (72.6–99.1)	B(8–9 y):90.8 (85.3–94.8)%	G: (4–5 y):43.8	G: (4–5 y):98.4
		T(4–5 y):0.898(0.816–0.979)		G(4–5 y):87.5% (47.4–97.9)	G(4–5 y):87.5% (77.6–94.1)	(6–7 y):38.1	(6–7 y):97.6
		T(6–7 y):0.915(0.824–1.00)		G(6–7 y):80% (44.4–96.9)	G(6–7 y):86.2% (77.5–92.4)	(8–9 y):31.6	(8–9 y):100
		T(8–9 y):0.950(0.926–0.974)		G(8–9 y):100% (81.3–100)	G(8–9 y):78.2% (71.4–84)		
5	Dong et al. (26)	B:	0.46	B:	B:	B:	B:
		(7–9 y):0.916(0.913–0.919)		7–9 y:100%	7–9 y:84%	(7–9 y):48%	(7–9 y):100%
		(10–12 y):0.909(0.905–0.912)		10–12 y:100%	10–12 y:82%	(10–12 y):53%	(10–12 y): 100%
		(13–15 y):0.950(0.946–0.955)		13–15 y:96%	13–15 y:94%	(13–15 y):74%	(13–15 y):99%
		(16–18 y):0.950(0.945–0.954)		16–18 y:97%	16–18 y:93%	(16–18 y):68%	(16–18 y): 100%
		T:0.932(0.930–0.934)		T:98%	T:88%	T:59%	T: 100%
		G:		G:	G:	G:	G:
		(7–9 y):0.926(0.919–0.933)		7–9 y:92%	7–9 y:93%	(7–9 y):66%	(7–9 y):99%
		(10–12 y):0.922(0.915–0.928)		10–12 y:89%	10–12 y:95%	(10–12 y):74%	(10–12 y):98%
		(13–15 y):0.926(0.920–0.933)		13–15 y:91%	13–15 y:94%	(13–15 y):70%	(13–15 y): 99%
		(16–18 y):0.920(0.915–0.926)		16–18 y:95%	16–18 y:90%	(16–18 y):59%	(16–18 y): 99%
		T:0.923(0.920–0.927)		T:92%	T:93%	T:67%	T: 99%
6	Ejtahed et al. (27)	B:	B:	B:	B:	NR	NR
		(7–10 y):93(91–95)	(7–10 y):0.50 (0.49–0.51)	(7–10 y):84(79–89)	(7–10 y):91(87–94)		
		(11–14 y):98(97–99)	(11–14 y):0.51 (0.50–0.53)	(11–14 y):94(91–97)	(11–14 y):93(89–95)		
		(15–18 y):98(97–99)	(15–18 y):0.51 (0.50–0.52)	(15–18 y):98(95–100)	(15–18 y):90(86–92)		
		(7–18 y):96(95–97)	(7–18 y):0.50 (0.49–0.52)	(7–18 y):93(90–96)	(7–18 y):90(87–92)		
		G:	G:	G:	G:		
		(7–10 y):95(94–97)	(7–10 y):0.50 (0.49–0.51)	(7–10 y):90(85–95)	(7–10 y):89(85–93)		
		(11–14 y):97(97–98)	(11–14 y):0.49 (0.49–0.50)	(11–14 y):97(94–98)	(11–14 y):90(88–92)		
		(15–18 y):98 (97–98)	(15–18 y):0.51 (0.50–0.52)	(15–18 y):95 (91–98)	(15–18 y):93 (89–96)		
		(7–18 y):97 (96–97)	(7–18 y):0.50 (0.49–0.500	(7–18 y):95(93–98)	(7–18 y):88(86–90)		
		T:	T:	T:88(84–92)	T:		
		(7–10 y):94(92–95)	(7–10 y):0.50 (0.49–0.50)	(7–10 y):88(84–92)	(7–10 y):89(85–91)		
		(11–14 y):97 (97–98)	(11–14 y):0.49 (0.49–0.50)	(11–14 y):97(95–98)	(11–14 y):89(87–90)		
		(15–18 y):98 (97–98)	(15–18 y):0.51 (0.51–0.52)	(15–18 y):96(93–97)	(15–18 y):92(89–94)		
		(7–18 y):96 (96–97)	(7–18 y):0.50 (0.49–0.51)	(7–18 y): 94–90–97)	(7–18 y):89(85–92)		
7	Fujita et al. (28)	B:0.981 (0.964–0.998)	B:0.519	B:100%	B:95%	NR	NR
		G:0.992 (0.981–1.004)	G:0.499	G:100%	G:95%		
8	Guntsche et al. (29)	Pubertal children: 0.99	B:0.54	Pubertal children: 97.2%	Pubertal children: 100%	NR	NR
		Prepubertal children: 0.98	G:0.54	Prepubertal children: 93.9%	Prepubertal children: 100%		
9	Kilinc et al. (3)	B:0.940	B:0.47	B:92.58%	B:78.97%	NR	NR
		G:0.907	G:0.46	G:90.05%	G:74.76%		
		Children: 0.926	Children: 0.49	Children: 89.51%	Children: 82.61%		
		Adolescents: 0.964	Adolescents: 0.46	Adolescents: 93.19%	Adolescents: 87.93%		
		T:0.920	T:0.47	T:89.89%	T:77.44%		
10	Kim et al. (30)	0.985(0.985–0.985)	0.48	97.6%	91.3%	54.7	99.7
11	Sousa et al. (20)	B:0.98 (0.97–1.00)	B:0.45	B:98.1% G:99.1%	B: 20.2% G:30.8%	NR	NR
		G:0.96 (0.95–0.98)	G:0.45				
12	Taylor et al. (31)	B:0.81 (0.73–0.90)	0.5	B:57%	B:76%	NR	NR
		G:0.79 (0.67–0.91)		G:74%	G:72%		
		T:0.79 (0.71–0.87)					
13	Zhou et al. (13)	B for central obesity: 0.983	B for central obesity: 0.47	B for central obesity: 94.3%	B for central obesity: 92.1%	NR	NR
		G for central obesity: 0.984	G for central obesity: 0.45	G for central obesity: 96.3%	G for central obesity: 90.5%		

AUC, area under the curve; PPV, positive predictive value; NPV, negative predictive value; WC, waist circumference; DXA, dual-energy X-ray absorptiometry; Y, year; B, boys; G, girls; T, total; NR, not reported.

### 3.5. Meta-analysis

Among 13 included studies in this systematic review, we could not pool data from five articles for the meta-analyses because we could not reach the authors for the data we needed, leaving eight articles to be included in the meta-analysis. Among the eight articles, four have indicated different overall cut-off points for diagnostic central obesity according to WHtR indicator, and eight have reported the cut-off values for both sexes.

**FIGURE 2:**
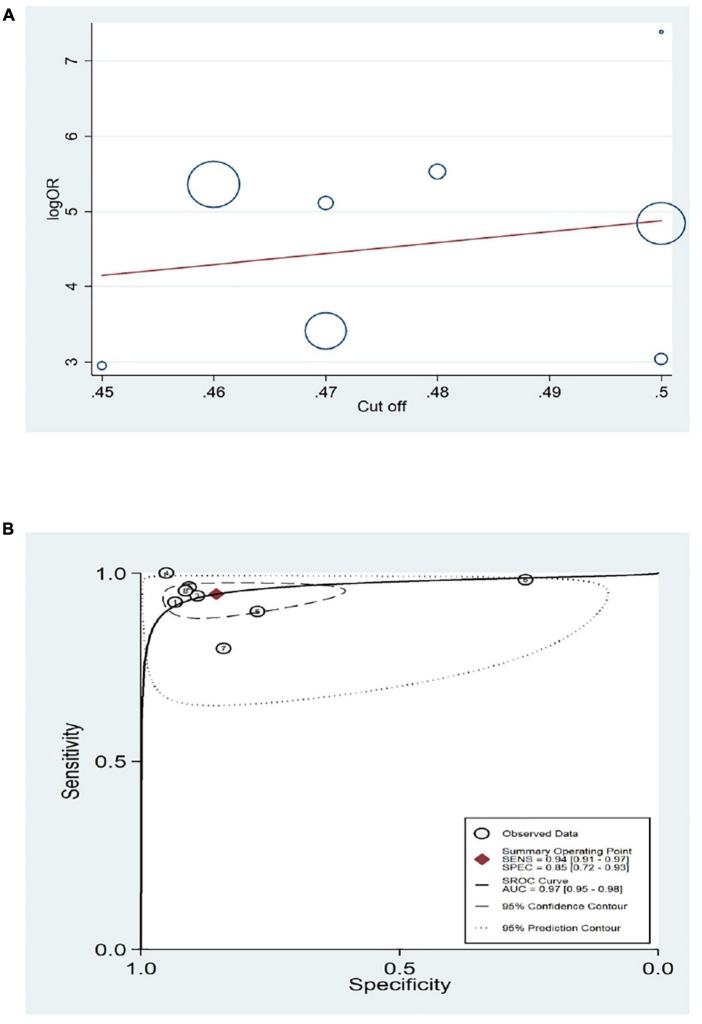
Diagnostic test accuracy meta-analysis for overall optimal cutoff score of WHtR for detecting central obesity in children and adolescence. **(A)** Regression lines of accuracy of WHtR for children and adolescence. **(B)** The optimal cutoff score 0.49 was marked as a cross in the estimated SROC curve.

**FIGURE 3:**
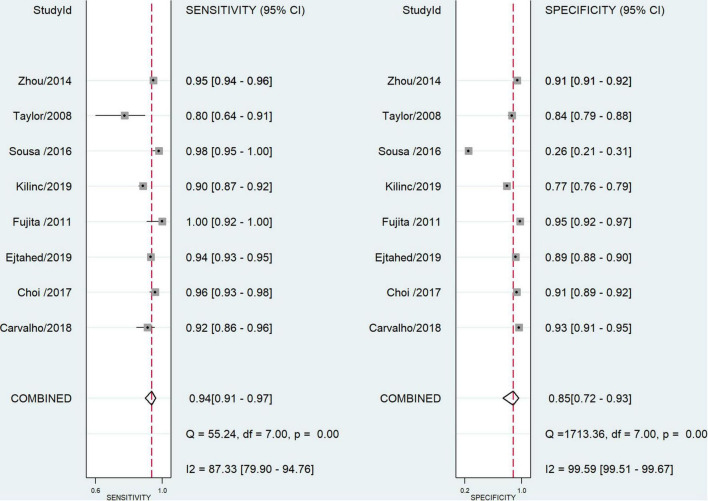
Forest plots for the diagnostic accuracy of overall cutoff point.

According to the Figure 2, the regression lines slopes show that accuracy of WHtR change with cut-off values, and across to characteristics of summary receiver operating characteristic curve (SROC curve), the optimal overall cut-off point was calculated at 0.49 (Figure 2). The pooled value of sensitivity and specificity of studies that have provided overall cut-off points were 0.94 (95% CI: 0.91–0.97) and 0.85 (95% CI: 0.72–0.93), respectively (Figure 3). The pooled PLR and NLR were 6.46 (95% CI: 3.23–12.93) and 0.07 (95% CI: 0.04–0.10), and the combined values of DOR was 99 (95% CI: 41.48–236.25). The studies heterogeneity according to pooled DOR was high (*I*^2^ = 100%). The forest plots were presented in a Supplementary material. Considering the cut-off score as a continuous variable, we carried out a multiple thresholds model to compute the optimal cut-off point of the WHtR index to detect visceral obesity in children and adolescents.

**FIGURE 4:**
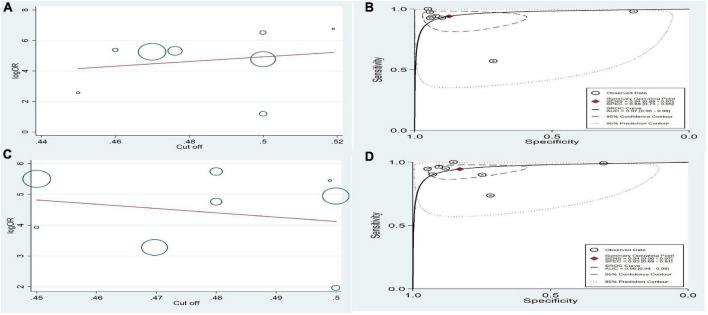
Diagnostic test accuracy meta-analysis for optimal cutoff score of WHtR for detecting central obesity in children and adolescence. **(A)** Regression lines of accuracy of WHtR for boys. **(B)** The optimal cutoff score 0.48 was marked as a cross in the estimated SROC curve. **(C)** Regression lines of accuracy of WHtR for girls. **(D)** The optimal cutoff score 0.49 was marked as a cross in the estimated SROC curve.

The pooled estimates sensitivity of cut-off points in girls and boys were 0.94 (95% CI: 0.90–0.97) and 0.94 (95% CI: 0.88–0.97), respectively. Also the combined specificity was 0.83 (95% CI: 0.69–0.91) in girls and 0.88 (95% CI: 0.73–0.95) in boys. We calculated pooled likelihood ratios in both sexes. The combined DOR in girls and boys were 83.2 (95% CI: 35.6–194.7) and 109.6 (95% CI: 29.6–405.9), respectively. The heterogeneity among the studies based on pooled DOR was high. The forest plots are shown in the Supplementary material. The optimal calculated cut-off scores to central obesity detection according to WHtR index was 0.49 and 0.49 in girls and boys, respectively (Figure 4).

### 3.6. Sensitivity analysis and publication bias

We performed sensitivity analyses by excluding the Taylor et al. study that was performed on 3–5 years children from the meta-analysis. To estimate the overall cutoff point, the summary sensitivity was 0.95 (95% CI: 0.92–0.97) and specificity 0.86 (95% CI: 0.70–0.94), and the optimal cutoff point was the same as the overall analysis. Additionally, for a finding of optimal cutoff points in different age ranges, the optimal cutoff point was calculated in studies with 10–18 years of participants, and the results of the analysis were similar to the overall cutoff score (Supplementary material).

We separately examined publication bias at the studies that had reported cut-off values in overall or both sexes. There was no asymmetry among the data points of the Deeks funnel plot of studies (*P* ≥ 0.05) (Supplementary material).

## 4. Discussion

To the best of our knowledge, the present study is the first systematic review and meta-analysis that summarized all evidence investigating the optimal cut-off value of WHtR for predicting abdominal obesity in children and adolescents of different ethnicities. The reported cut-off values and their sensitivities and specificities were collated to provide a universal, practical, and accurate criterion for screening central obesity.

Totally, thirteen articles were included in this systematic review and meta-analysis was done on eight articles. We reached the number 0.49 as the optimum cut-off value for boys, girls and overall to predict central obesity in children and adolescents. Our findings confirmed that the same cut-off value can be used for both sexes. The maximum and minimum of the reported cut-off values among the included studies were 0.54 for Argentina (29) and 0.45 for Brazil (20), respectively. The variance in optimal reported cut-offs between studies may be due to differences in races and ethnicities (10). In a newly published systematic review and meta-analysis, different WHtR cut-off values were reported for populations of children and adolescents with different ethnicities as an indicator of cardiometabolic risks (21). The calculated optimal cut-off for East and Southeast Asian region and Latin American region was 0.46 and 0.54, respectively (21). Moreover, it should be noted that measurement of the indices like waist circumference and height depends on the used protocols which may cause variability of the cut-offs between different studies. With regard to a recent systematic review, the weighted average WHtR cut-off points of 0.47 and 0.46 have been reported to predict central obesity in 6–18 years old boys and girls, respectively (33). Another systematic review and meta-analysis which published in 2021 evaluated the performance of the WHtR for identifying cardio-metabolic risks in children and adolescents and reported high heterogeneity regarding the optimal cut-off of WHtR among different ethnicities (34). In this systematic review, we have not included the studies that evaluated pre-defined WHtR cut-off (0.5). According to the NICE guideline, WHtR range of 0.4 to 0.49 indicates healthy central obesity and without increased health risk, but WHtR 0.5 and more indicates increased health risks (35). However, our final cut-off value is approximately equal to the universally accepted one (7, 36) which is certainly easier to memorize and utilize. Practitioners, parents, care-givers or youth themselves can measure the index just with a string with no need for tape.

In this systematic review and meta-analysis, the pooled sensitivity of optimal cut-off point (0.49) in girls and boys were 0.94 and 0.94, respectively. Also the combined specificity was 0.83 in girls and 0.88 in boys. In recent meta-analysis, the sensitivity and specificity of WHtR performance in screening central obesity in children and adolescents have been reported as 0.91 and 0.90, respectively (25). Besides, several studies aimed to investigate the sensitivity and specificity of the pre-defined cut-off point of 0.5 in their sample population. In one study, sensitivity of 91% and specificity of 95% were reported in Greek adolescents (37). Another study was carried out on 649 American children (2–18 years) and proposed 99 and 72% for sensitivity and specificity of pre-defined cut-off point of WHtR in predicting central obesity (38).

The pooled calculated AUC for our suggested cut-off value (0.49) is 0.96, which proves we can predict childhood central obesity with high accuracy. Our pooled AUC was very close to the previous meta-analysis which reported AUC = 0.96 for WHtR (34). Another systematic review and meta-analysis assessed the discriminatory capacity of the anthropometric indices for body fat and revealed an excellent power of WHtR in males (AUC: 0.897) and females (AUC: 0.914) (39).

In our study the pooled estimated DOR of WHtR to predict central obesity was 102 (95% CI: 50–207). This finding was concordant with pervious study which reported that DOR of WHtR for predicting enteral obesity was 88 (95% CI: 40–195) (25). A little discrepancy between our estimated DOR with that study was due to this point that we estimated DOR according to our pooled estimated optimal cut-off point (0.49).

Different anthropometric indices with different strengths and limitations are used to diagnose childhood obesity. BMI and WC are the most commonly used indices as a screening tool for obesity worldwide. However, BMI cannot differentiate fat mass (33). On the other hand, WC is another anthropometric index used to diagnose central obesity. It should be noted that age and sex-specific curves are required for both indices in clinical practice. Recent studies have proposed WHtR as a new anthropometric indicator facilitating the diagnosis of obesity, specifically central obesity in children and adolescents. As evidenced in our study, WHtR is less dependent on age and sex and does not need charts for interpretation. Moreover, WHtR has the superiority of predicting health risks related to central obesity such as type 2 diabetes, hypertension, and cardiovascular disease in children and adolescents aged five and more (35, 40). Several studies have shown that WHtR had the largest discriminatory power for metabolic disorders such as diabetes and dyslipidemia in comparison with WC, BMI and waist-to-hip ratio (41, 42). A cross-sectional study evaluated the usefulness of the WHtR in predicting cardiometabolic risks in children in five European countries. They suggested WHtR > 0.55 as an appropriate boundary value for screening young European population at high cardiometabolic risk (43). The higher WHtR value in children and adolescents could predict high cardiometabolic risks in future life (44–46).

### 4.1. Strengths and limitations

The main strength of this study is that we comprehensively reviewed 13 studies and analyzed data of a large number of participants (total sample size = 180,119) and proposed an optimum cut-off value of WHtR for diagnosis of central obesity with high accuracy in children and adolescents which verify that approximately the predefined cut-off 0.5 is appropriate cut-off value in clinical setting. Moreover, some of the included studies were from national surveys. This study has some limitations. Thirteen studies met the inclusion criteria for this systematic review. However, we could not access to the required data of five articles for the meta-analysis despite our efforts to contact the researchers (23, 25, 26, 29, 30); hence, eight articles were used in the analysis. Also, since the main goal of this study was to estimate the optimal cut-off value, therefore we excludes studies which assessed sensitivity and specificity, AUC, DOR according to pre-defined cut-off (0.5). This exclusion criteria which we considered in our study may effect on the pooled estimated of these diagnostic criteria.

## 5. Conclusion

The results of the current systematic review and meta-analysis confirm that WHtR cut-off value could predict central obesity with high accuracy in children and adolescents with various races, ages, and genders. Although 0.49 is the proposed theorical cut-off value, 0.5 is much more practical value in children and adolescents. Moreover, it can be easily communicated with the message “keep your waist to less than half your height” (35). Totally, it is recommended to use WHtR cut-off value as a simple tool to screen central obesity without the need for any charts in practice.

## Data availability statement

The original contributions presented in this study are included in this article/Supplementary material, further inquiries can be directed to the corresponding authors.

## Author contributions

MQ and H-SE came with the idea of this manuscript. FP and ME did the study search, evaluated the articles, wrote the manuscript, and extracted the data then prepared the tables. MK did the meta-analysis and wrote statistical analysis method and result section. JT reviewed the manuscript. ZE-A and KP did the final required revisions. MQ and H-SE supervised all the process and did the final proof of the manuscript. All authors contributed to the article and approved the submitted version.

## Supplementary material

Supplementary Figure 1 Forest plots of NLR values on overall cut-off point.

Supplementary Figure 2 Forest plots of DOR values on overall cut-off point.

Supplementary Figure 3 Forest plots for the diagnostic accuracy of cutoff point in girls.

Supplementary Figure 4 Forest plots of NLR values on cut-off point in girls.

Supplementary Figure 5 Forest plots of DOR values on cut-off point in girls.

Supplementary Figure 6 Forest plots for the diagnostic accuracy of cutoff point in boys.

Supplementary Figure 7 Forest plots of NLR values on cut-off point in boys.

Supplementary Figure 8 Forest plots of DOR values on cut-off point in boys.

Supplementary Figure 9 Deek’s funnel plot analysis **(A)** cutoff point in girls **(B)** cutoff point in boys.
