# Supplementary figures and images for: Optimal cut-off value of waist circumference-to-height ratio to predict central obesity in children and adolescents: A systematic review and meta-analysis of diagnostic studies
Source: Front Nutr. 2023 Jan 4;9:985319. doi: 10.3389/fnut.2022.985319 (PMC9846615; doi:10.3389/fnut.2022.985319)

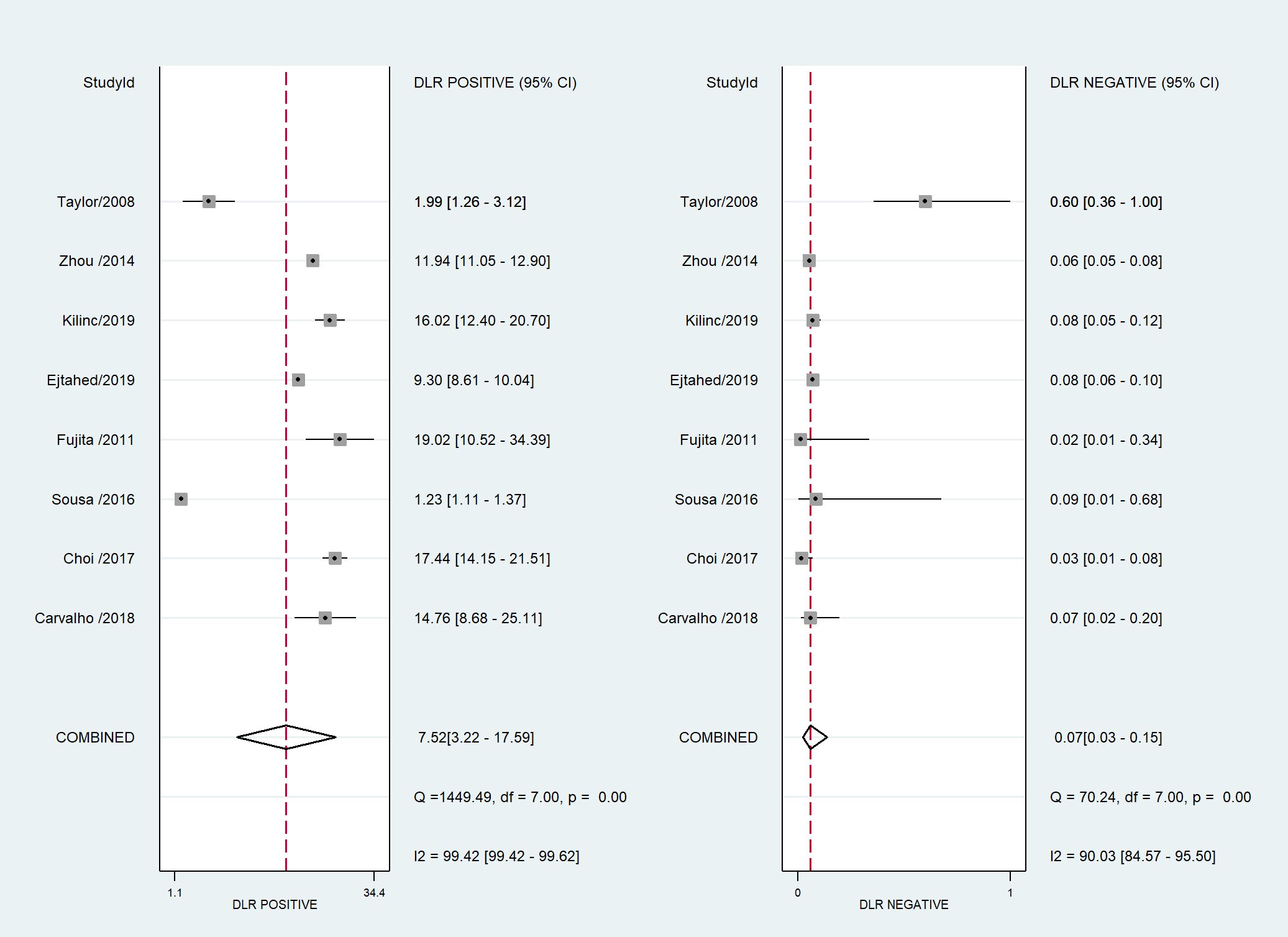

Supplement: Supplementary Figure 1 — Forest plots of NLR values on overall cut-off point. [file Image_1.JPEG]

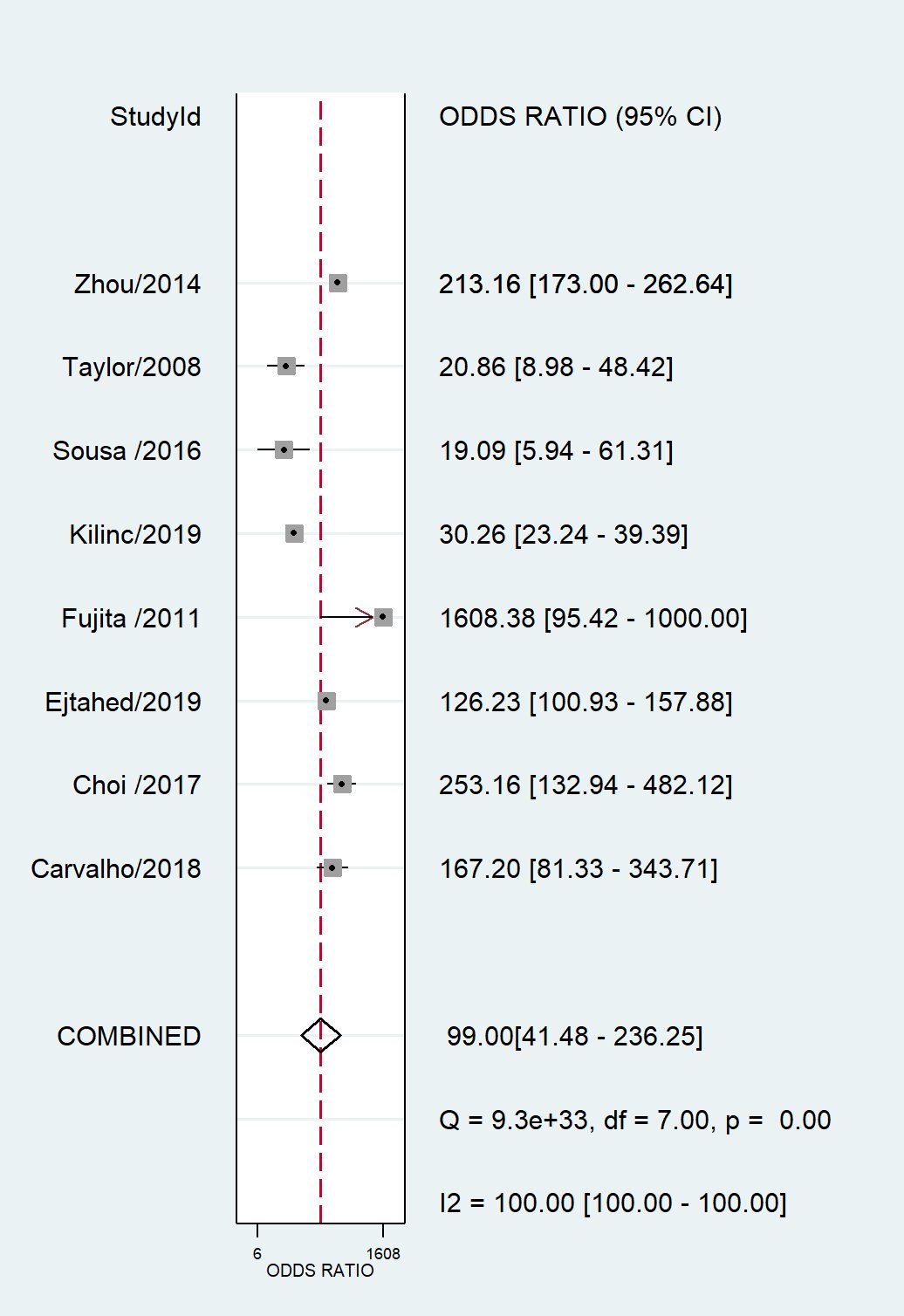

Supplement: Supplementary Figure 2 — Forest plots of DOR values on overall cut-off point. [file Image_2.JPEG]

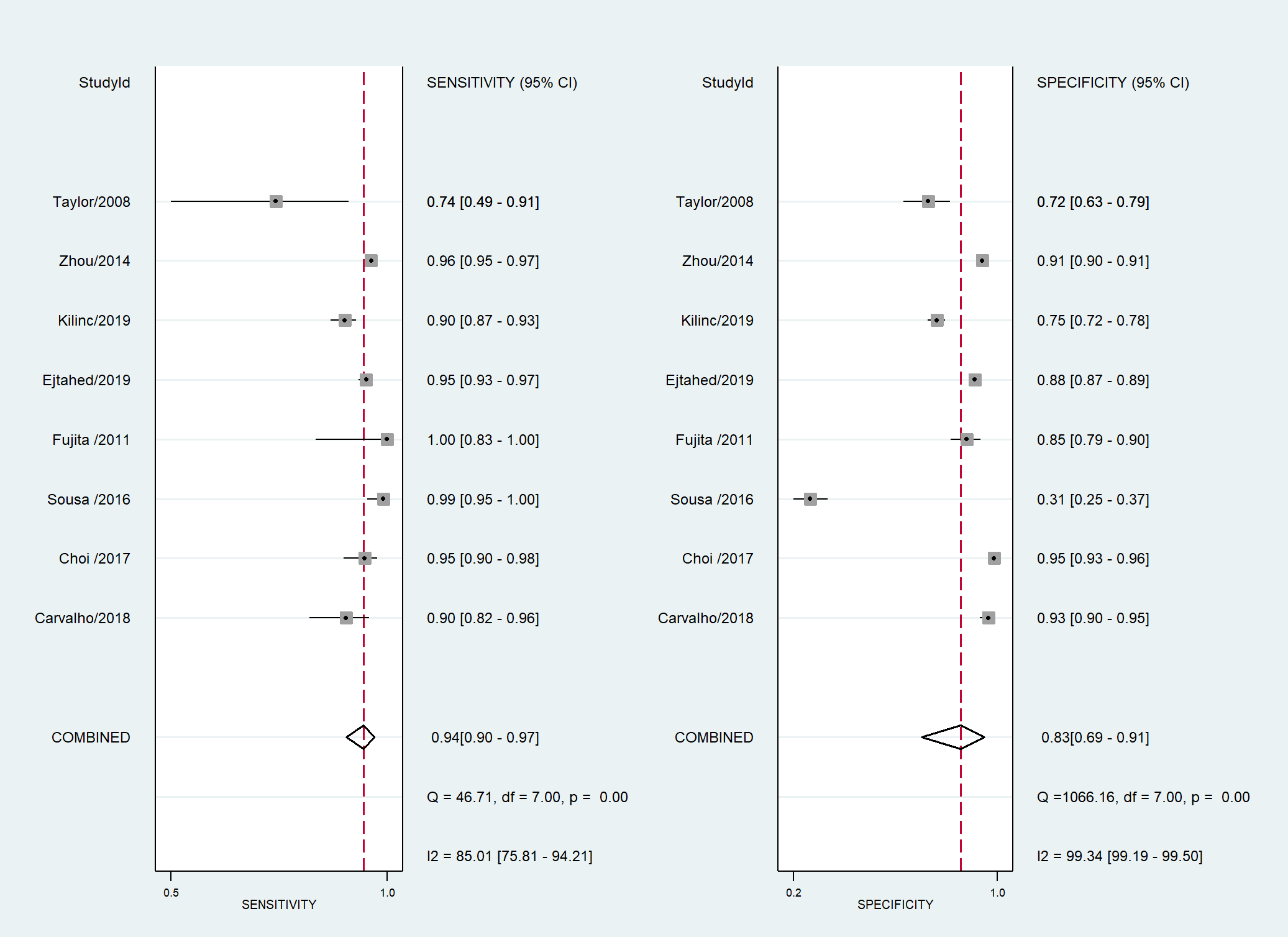

Supplement: Supplementary Figure 3 — Forest plots for the diagnostic accuracy of cutoff point in girls. [file Image_3.TIF]

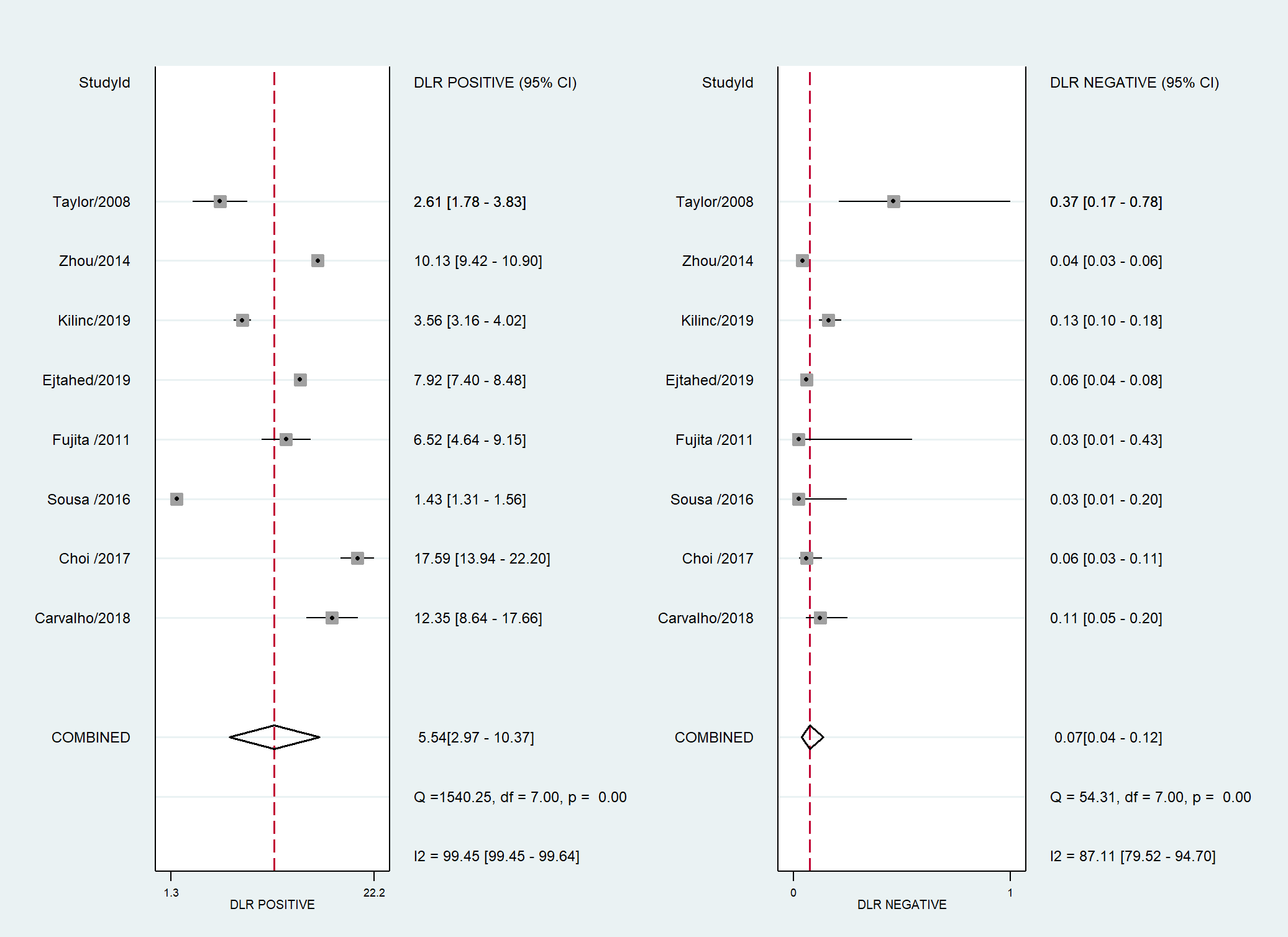

Supplement: Supplementary Figure 4 — Forest plots of NLR values on cut-off point in girls. [file Image_4.TIF]

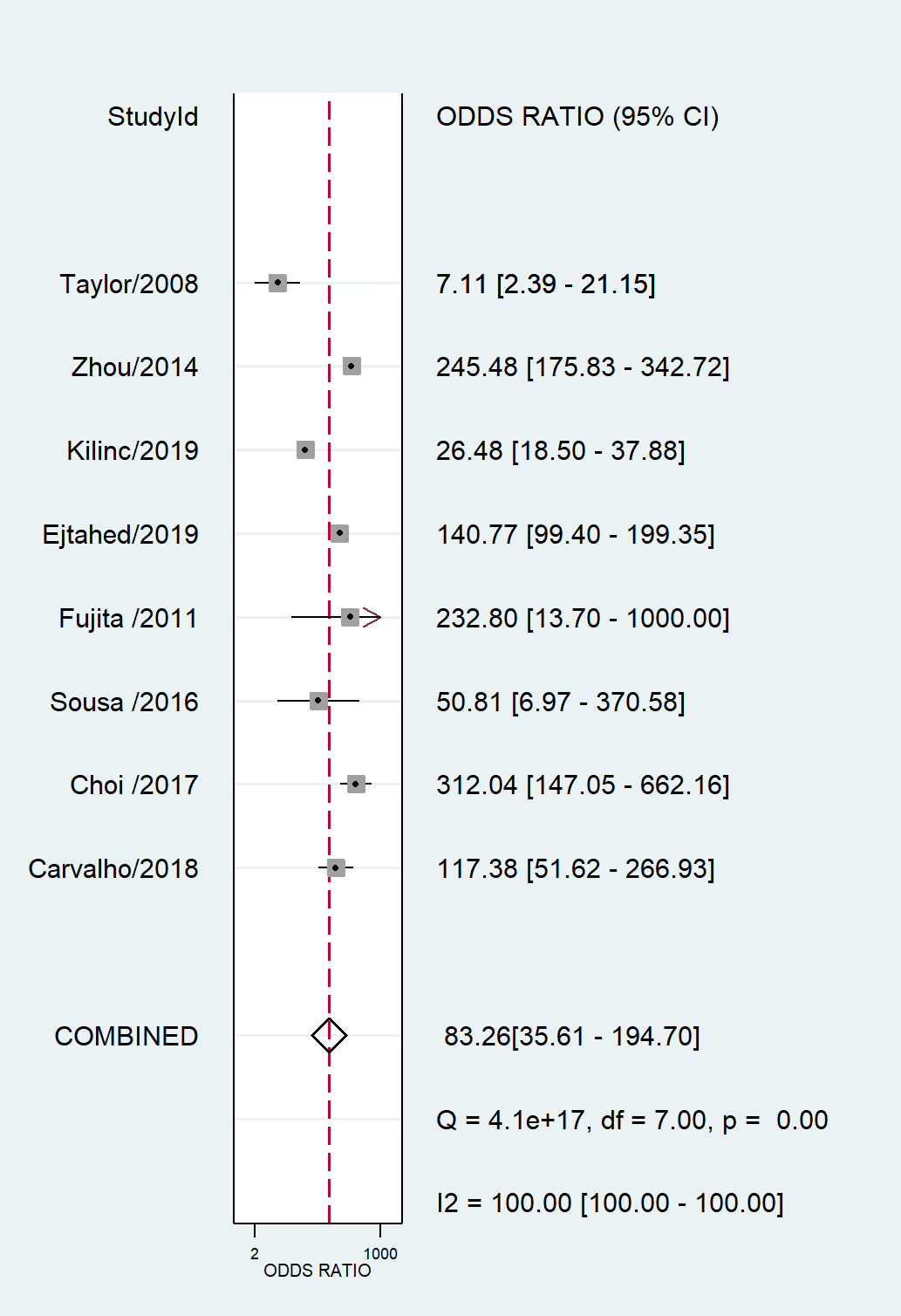

Supplement: Supplementary Figure 5 — Forest plots of DOR values on cut-off point in girls. [file Image_5.TIF]

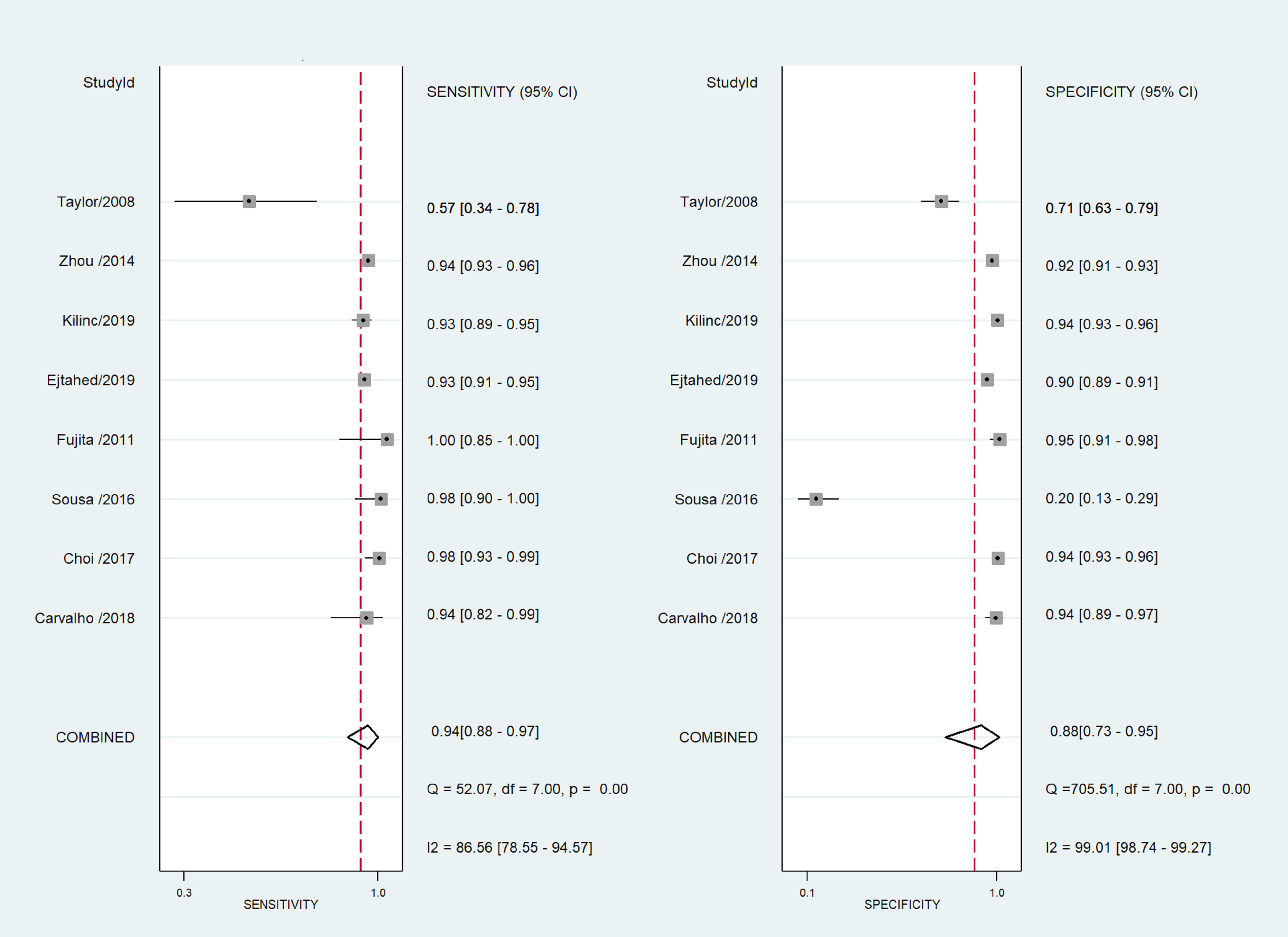

Supplement: Supplementary Figure 6 — Forest plots for the diagnostic accuracy of cutoff point in boys. [file Image_6.TIF]

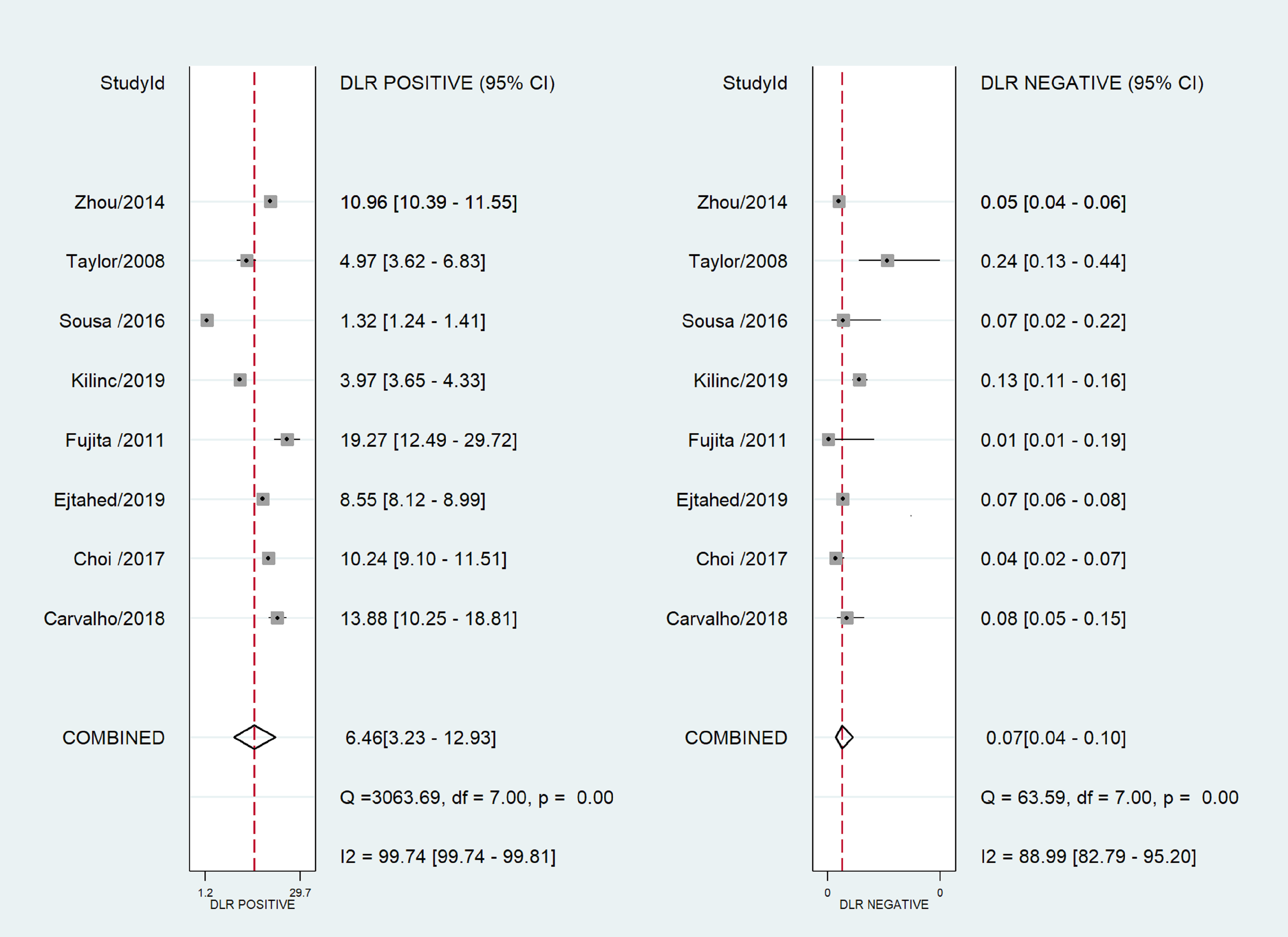

Supplement: Supplementary Figure 7 — Forest plots of NLR values on cut-off point in boys. [file Image_7.TIF]

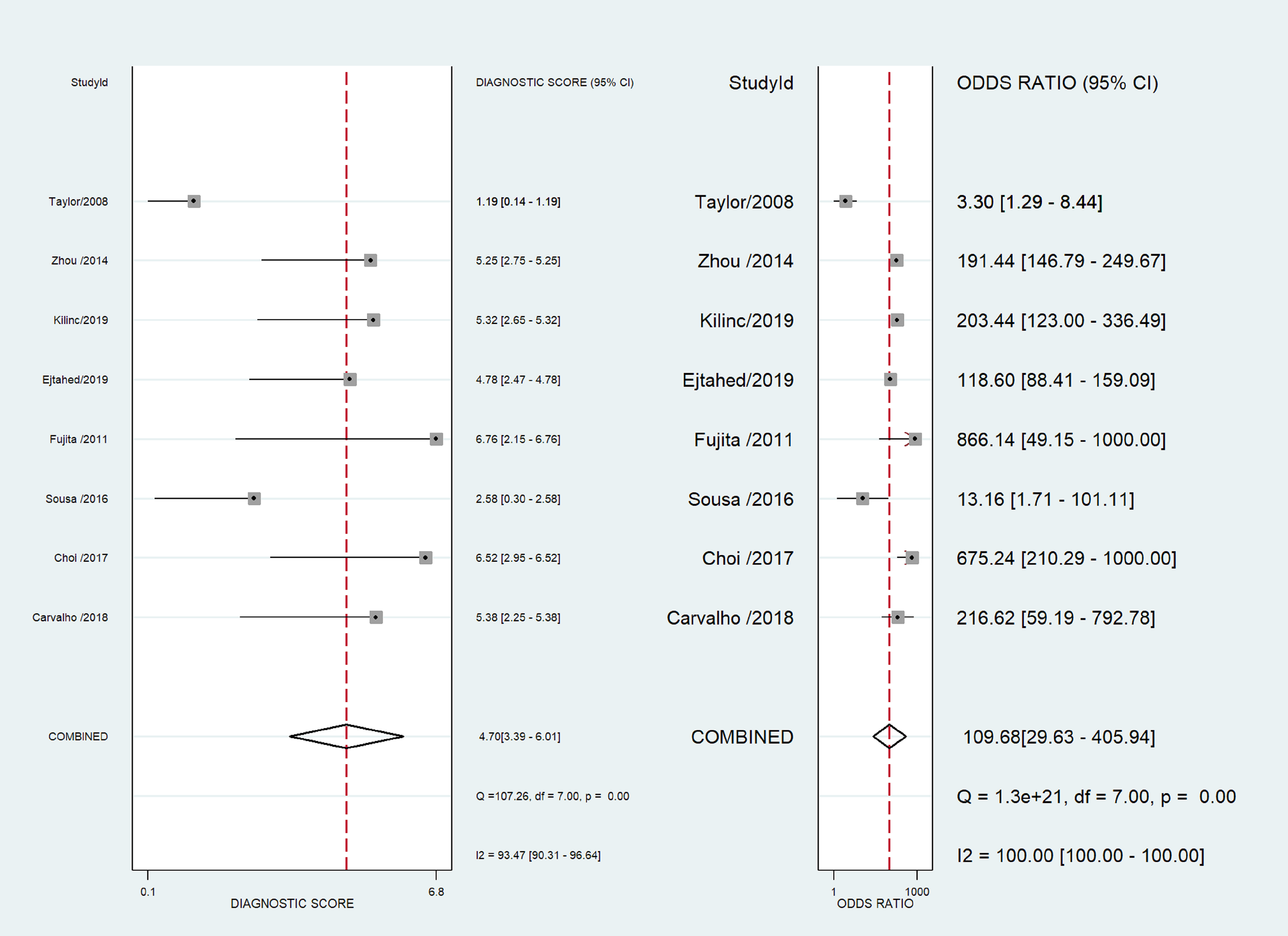

Supplement: Supplementary Figure 8 — Forest plots of DOR values on cut-off point in boys. [file Image_8.TIF]

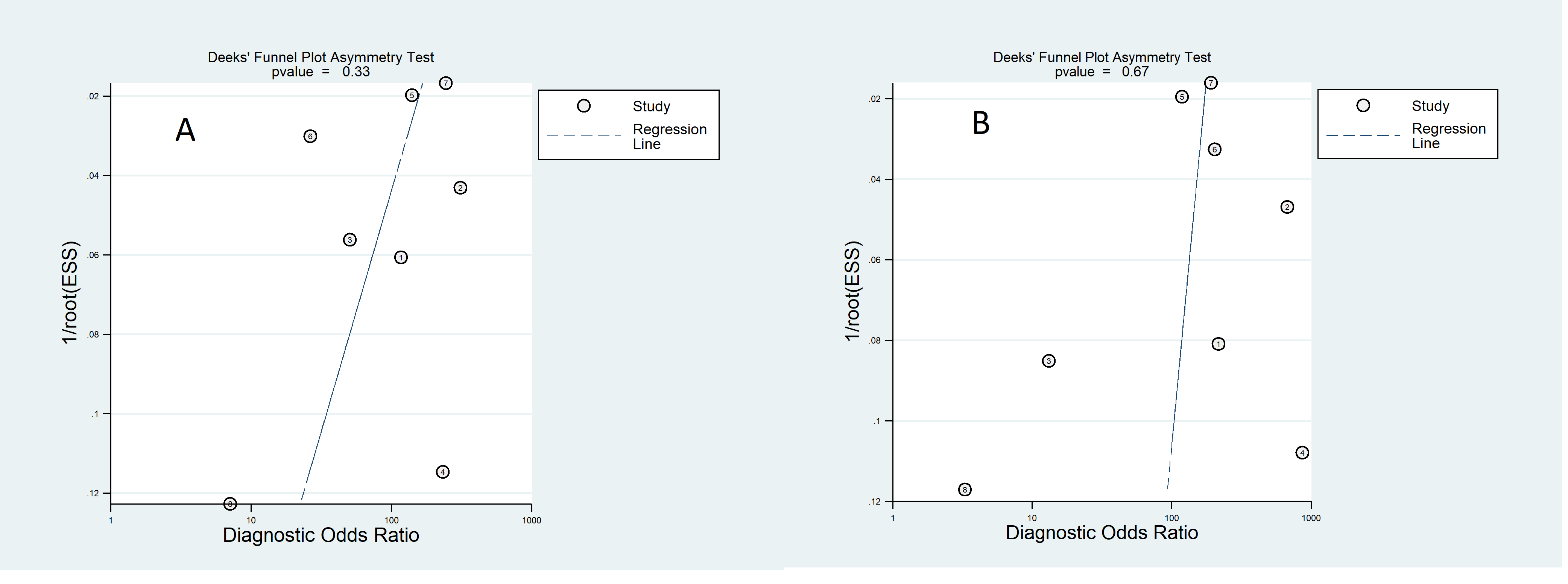

Supplement: Supplementary Figure 9 — Deek’s funnel plot analysis (A) cutoff point in girls (B) cutoff point in boys. [file Image_9.PNG]
